# Genetic Engineering and Synthetic Genomics in Yeast to Understand Life and Boost Biotechnology

**DOI:** 10.3390/bioengineering7040137

**Published:** 2020-10-29

**Authors:** Daniel Schindler

**Affiliations:** 1 Manchester Institute of Biotechnology, The University of Manchester, 131 Princess Street, Manchester M1 7DN, UK; 2 Max Planck Institute for Terrestrial Microbiology, Karl-von-Frisch-Straße 10, 35043 Marburg, Germany; daniel.schindler@mpi-marburg.mpg.de; Tel.: +49-6421-178533

**Keywords:** genetic engineering, synthetic biology, *Saccharomyces cerevisiae*, synthetic genomics, Sc2.0, Sc3.0, yeast, biotechnology, cell factory

## Abstract

The field of genetic engineering was born in 1973 with the “*construction of biologically functional bacterial plasmids in vitro*”. Since then, a vast number of technologies have been developed allowing large-scale reading and writing of DNA, as well as tools for complex modifications and alterations of the genetic code. Natural genomes can be seen as software version 1.0; synthetic genomics aims to rewrite this software with “build to understand” and “build to apply” philosophies. One of the predominant model organisms is the baker’s yeast *Saccharomyces cerevisiae*. Its importance ranges from ancient biotechnologies such as baking and brewing, to high-end valuable compound synthesis on industrial scales. This tiny sugar fungus contributed greatly to enabling humankind to reach its current development status. This review discusses recent developments in the field of genetic engineering for budding yeast *S. cerevisiae,* and its application in biotechnology. The article highlights advances from Sc1.0 to the developments in synthetic genomics paving the way towards Sc2.0. With the synthetic genome of Sc2.0 nearing completion, the article also aims to propose perspectives for potential Sc3.0 and subsequent versions as well as its implications for basic and applied research.

## 1. Introduction

“Transformation” of an organism was reported for the first time almost 100 years ago: in 1928, Frederick Griffith reported he had changed the type of one *Streptococcus pneumoniae* to another [1]. At this time, Griffith was not aware that the “substance” which was transforming a living organism was actually deoxyribonucleic acid, or in short: DNA. The predominant opinion at this time was that proteins were responsible for transformation, but in 1944, researchers identified the transformative “substance” to be DNA [2]. The next milestone in this regard was the development of in vitro cloning, which earned Paul Berg the 1980 Nobel Prize for his work studying the fundamental biochemistry of nucleic acids, particularly recombinant DNA [3]. Paul Berg shares the Nobel Prize with Walter Gilbert and Frederick Sanger for contributions to early nucleic acid sequencing. This award already demonstrated around 40 years ago that the “reading” and “writing” of DNA go hand in hand and advance their technological boundaries, respectively.

The field of genetic engineering was born with the work of Stanley Cohen and Herbert Boyer with the first report of the “construction of biologically functional bacterial plasmids in vitro” [4]. From this starting point, numerous research fields were developed, which are not easy to define due to their overlap. For the purposes of this review, the term genetic engineering refers to any application of changing the genetic code of an organism with molecular biology tools, and biotechnology is a branch of its industrial application. Genetic engineering covers the rising disciplines of synthetic biology*,* which takes a rational engineering approach for the construction of genetic material, and synthetic genomics, where whole chromosomes and genomes are chemically synthesised and replace their natural counterparts. In this review, xenobiology is also considered to be genetic engineering which uses non-standard and non-natural building blocks in addition to the natural building blocks of life within an organism. All of the aforementioned research fields have overlapping areas and their respective developments benefit and influence one another.

Transitioning from the discovery and utilisation of recombinant DNA to the current state-of-the-art in genetic engineering required many key developments and, alongside *Escherichia coli*, another model organism which is essential for our current abilities in genetic engineering is *Saccharomyces cerevisiae*. Key factors in the rise of *S. cerevisiae* as a model organism, besides being easy to handle and its generally recognized as safe (GRAS) status, were the first transformation of yeast, the discovery of the highly efficient homologous recombination machinery and its being the first eukaryote with a fully sequenced genome [5,6]. Sequencing technologies have been developed over recent years. Initially, sequencing was performed on a single DNA fragment at a time and this strategy is still used in the laboratory routine to confirm shorter individual DNA sequences [7,8]. These first-generation sequencing strategies have been gradually replaced by next-generation sequencing (NGS) methods, where millions of molecules with relatively short read lengths are sequenced at the same time and subsequently assembled into a longer consensus sequences via respective computer programs [9,10]. The availability of a huge number of reference genomes allows for low coverage sequencing and comparison with databases to provide meaningful results at low cost [11]. Interestingly, in recent years, new sequencing technologies, called third-generation sequencing [12], have emerged which combine the advantage of long sequencing reads and the massive parallel sequencing of the second generation, but come with the drawback of a lower accuracy [13,14]. In particular, nanopore sequencing seems to have established itself in many laboratories based on the rather low investment in equipment and its tiny size—calling a nanopore sequencer a benchtop machine would be an exaggeration and is highlighted by the ability to use the machine in the field in highly remote environments [15,16]. Based on identified genes, we are able to decode the networks of life and to identify pathways which can produce highly valuable compounds—one of the most popular ones might be penicillin, an antibiotic which was a game changer in modern medicine and can be produced in yeast [17,18]. The broad application of NGS and industrial competition were some of the driving forces to reduce sequencing costs [19]. A similar effect based on competition and emerging technologies can be observed for DNA synthesis [20]. DNA synthesis is still largely based on chemical oligonucleotide synthesis by the phosphoramidite method but enzymatic DNA assembly is starting to invade the market. Enzymatic DNA synthesis has the potential to become a game changer for DNA writing, similar to NGS for DNA reading and will likely change the price per base pair to allow the synthesis of the building blocks for an entire synthetic *E. coli* genome for USD 10,000 (~USD 0.0025 per bp). Besides the drop in costs for DNA synthesis, the development of DNA sequence design tools allows researchers to scale up their experimental designs, allowing construction and automated manipulation on the whole chromosome and genome level [21,22]. The recent developments in DNA reading and writing allow researchers now to transition to the phase of creative genome writing without being a software engineer.

The genetic code could be interpreted as a type of software, and the wild type or natural genome could be considered as version 1.0. Extensive work in genetic engineering has allowed researchers to make small or large changes to the genetic code to understand life and generate valuable production strains, such as a yeast strain producing a human hepatitis B vaccine [23]. One of the most important developments besides in vitro DNA cloning has been the development of polymerase chain reaction (PCR) which allows for the recovery of any DNA sequence in any required amount [24]. The development of PCR laid the path for the extensive genome engineering possible today and is the basis of certain strategies to generate long, chemically synthesised DNA fragments. These key developments in tools and the ability to read genomes allow researchers to now rewrite complete genetic codes on the chromosome and even whole genome level. Synthetic genomes such as those of the microorganisms *Mycoplasma mycoides*, *E. coli* and the yeast *S. cerevisiae* are the first examples [21,25,26,27]. Current “writing” projects have been until now mostly “rewriting” of the original code with minor changes and can be considered as genome version 2.0. However, technological developments and the resulting price drops in DNA synthesis will facilitate “creative writing” efforts and a deeper understanding of life as well as a boost for biotechnology and can potentially result in genome 3.0 versions and beyond [20,27,28].

This review focuses on the new generation of genetic engineering concepts in *S. cerevisiae* which are used to understand the fundamentals of life and to unleash biotechnology. It will be based on genetic engineering in natural and laboratory wild type strains—considered Sc1.0. An international consortium is nearing the completion of the first fully synthetic eukaryote: *S. cerevisiae* 2.0 (Sc2.0). The consortium has already shown in current semi-synthetic strains what the potential of fully synthetic yeast strains might be [29]. Besides this, the article will give perspectives on how lessons from Sc1.0 and Sc2.0 could result in a yeast Sc3.0. It is important to bear in mind that discoveries and technological developments generated in *S. cerevisiae* are driving forces to be able to engineer non-conventional and non-model organisms. It has also been shown that findings in yeast can be transferred to humans and *S. cerevisiae* can even be applied as a model for human diseases. Furthermore, Sc2.0 is the driving force to initiate such ambitious projects such as the Genome Project-write (GP-write), which ultimately aims to generate stable and safe cell lines [30].

## 2. Yeast 1.0 Evolution to Become a Fundamental Model Organism

Yeasts, not only *S. cerevisiae*, are undeniably one of the most influential and valuable model organisms. Many discoveries and applications have been made or are based on these unicellular fungi. Unknowingly, the abilities of yeast to perform fermentation have been harnessed by humans for thousands of years. Humankind would not have such a cultural heritage if yeast had not performed the miracles of ancient biotechnology, giving humans bread and various types of widely loved beverages. The complex history of yeast can be highlighted with the statement that humans have been performing biotechnology for more than 5000 years [31]. Yeast is potentially the second most important domestication humans achieved, right after the domestication of fire! However, only since 1680 were we aware that yeast is present in beer. This discovery was made by Antonie van Leeuwenhoek, who is considered to be the father of microbiology with his passion for microscopic observations. It took almost two additional centuries until Louis Pasteur demonstrated yeast as being responsible for the production of alcohol via fermentation in 1858 [32]. Yeast became increasingly important in research, and in 1988, yeast was proposed as “an experimental organism for modern biology” [33] and it still is “an experimental organism for 21st century biology” [34]. Notably, around 23% of yeast genes have homologues in the human genome, which allows for the transfer of ground-breaking research findings to humans. This might also be one of the reasons why many Nobel Prizes have been awarded for performing research with yeast as a model [35].

It is therefore unsurprising that the *S. cerevisiae* genome was the first eukaryotic genome which was fully sequenced. The rather simple genome structure, point centromeres, comparably low numbers of complex repetitive sequences and the ability to be cultured as a haploid organism are just some facets of this extraordinary organism. Yeast might be the eukaryotic model organism with the largest amount of different collections, ranging from gene deletions and depletion collections towards localisation and expression collections and flexible collections, which allow the user to repurpose the original collection for their respective interests.

## 3. Yeast 1.0 as a Cell Factory to Produce Valuable Molecules

The “classical” use of yeast for the production of the daily goods of joy without any genetic engineering, initially unknowingly, made yeast a cell factory. With the dawn of genetic engineering, yeast became a rising star as a cellular factory in biotechnology. Each of these fields rely on the transformation of yeast cells with recombinant DNA, which is either integrated into the genome or maintained as an additional plasmid with the goal of obtaining a valuable product. The product range produced in yeast is broad and constantly increasing. Interestingly, often initial research in yeast is later transferred to “non-conventional” yeast species or other organisms to boost the production [36,37].

**Figure 1:**
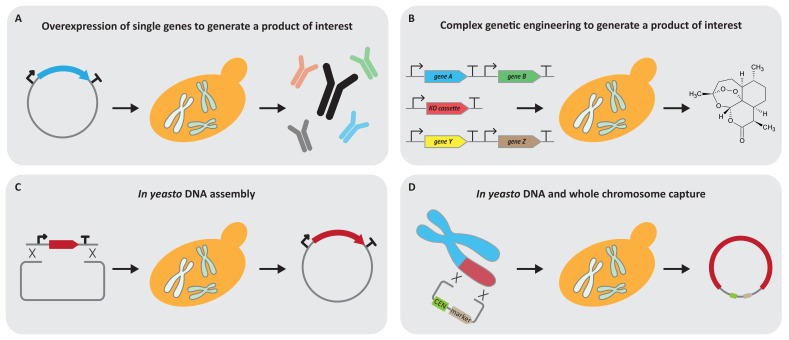
Established yeast 1.0 applications. (**A**) Yeast has been widely used to overexpress single genes which result in a specific product which can be either a protein or a valuable substance. (**B**) The field of genetic engineering quickly evolved and allows complex genetic modifications including, but not limited to, the introduction and/or deletion of genes to optimise yeast as a cell factory for the intended purpose. (**C**) Yeast also has established itself as a DNA assembly host. *In yeasto* cloning has been used to construct individual plasmids and even whole chromosomes. (**D**) The genome plasticity and ability of DNA uptake of yeast are remarkable, and allow the yeast to capture large DNA fragments up to whole chromosome size under laboratory conditions. Once this DNA is in yeast, it can be modified with the extensive genetic engineering toolbox of yeast.

Yeast has widely been used for the expression and production of valuable products ranging from the expression of single genes and smaller pathways towards the field of metabolic engineering, which rewires the network of a cell extensively to optimise fluxes of metabolites within a cell to boost the production of the desired compound (Figure 1A,B). Yeast has become a platform for a broad range of products from antibiotics to fine chemicals to petrochemicals and vaccines [38,39,40]. Large-scale projects, for example, achieved the production of artemisinin and the synthesis of opioids in yeast [41,42]. In a recent study in the laboratory of Christina Smolke, yeast has been modified to produce tropane alkaloids, which are valuable compounds for medical applications [43]. This yeast platform contains 15 additional genes and seven gene disruptions. The study served as the foundation for a more recent publication to produce the medically relevant alkaloids hyoscyamine and scopolamine in yeast [44]. In total, 34 chromosomal modifications were performed and, besides the deletion and addition of genes, subcellular protein localisations for the respective reactions were taken into account. This might currently be the most complex genetic engineering approach to produce substances in yeast.

Interestingly, the ability to make yeast a cell factory is also extended to non-laboratory strains, which may one day have an application in the food industry. In a recent study, Jay Keasling and colleagues engineered industrial brewing yeasts for some of the primary flavour determinants in hopped beer [45]. Such industrial strains would make beer production more sustainable, as hops are water- and energy-intensive crops and their flavour-determining compounds vary between harvests. Applying yeast as a biotechnological chassis has more potential sustainable applications beyond “just” the production of petrochemicals as sustainable biofuels. However, one should always keep in mind that extensive engineering of a given chassis can potentially induce burden on cells [46,47]. These chassis are alive and therefore are underpinned by the principles of life, which include evolution; production strains may not be stable over time, which is an issue when initial experiments are scaled up to industrial sizes [48].

All mentioned examples benefit from the extensive work which has been performed in *S. cerevisiae*, resulting in a massive toolbox for genetic engineering. The increasing “industrialisation” of molecular biology and technical advances allow researchers to scale up studies, resulting in automated laboratories and the generation of biofoundries to be able to cope with the scale of projects [49]. In recent years, yeast has become an organism which is not only used to produce valuable products, it is increasingly hijacked to build DNA fragments based on its highly efficient homologous recombination machinery. The dimensions of building DNA in yeast are now in the megabase range and allow for the construction of designer chromosomes and whole bacterial genomes [27].

## 4. Yeast 1.0 as an Engineering Platform to Build and Manipulate DNA Up to Whole Chromosomes

Homologous recombination of DNA in yeast is highly efficient and non-homologous end joining of DNA is a rather rare event. Yeast only needs 20 bps of homologous DNA sequence close to the ends of a DNA fragment to accurately paste it into the genome [50]. This intrinsic ability of *S. cerevisiae* has allowed for the construction of numerous resources, such as deletion collections. Researchers have hijacked the homologous DNA repair pathway to build plasmids at low cost and in a high-throughput manner [51,52,53] (Figure 1C). Many technologies have evolved, such as transformation-associated recombination (TAR) cloning, which allows for the capture of large heterologous DNA fragments [54] (Figure 1D). Amazingly, yeast seems to have an enormous genome plasticity and uptake of DNA on the megabase scale has been demonstrated [55,56]. Strikingly, only minor changes in bacterial genomes allow for the transfer of whole chromosomes into yeast [57]. Furthermore, advances in DNA synthesis allow for the construction of whole synthetic chromosomes and genomes in yeast [27]. Yeast has established itself as the workhorse to allow for this large-scale engineering. The phrase “DNA assembly *in yeasto*” has become a popular alternative for “in vivo homologous recombination-based DNA assembly in yeast” and shows the establishment of yeast for large-scale DNA assembly [58].

Two different approaches have been established in the area of heterologous genome engineering in yeast: one is the capturing of an existing genome in a yeast cell and its maintenance as an episomal vector [57,59,60,61,62,63,64]. The other, which is more work intensive, is the construction of chromosomes from amplified or synthesised DNA fragments as episomal vectors [25,65,66,67]. Both strategies have in common that once the episomal vector is integrated into the yeast nucleus, it is maintained as an additional “circular chromosome” with the respective yeast centromere and at least one autonomously replicating sequence (ARS). Once in yeast, the constructs can be modified and altered with the large and constantly increasing genetic engineering toolbox [68,69,70]. Genome capturing has an obvious advantage that once the minimal requirements to propagate DNA in yeast are integrated into the DNA, there is a chance that it can be transferred into yeast and be stably maintained. This allows for the modification of genomes which are not usually accessible on a low-cost scale. However, if large modifications are necessary, for example, codon replacements on a whole genome level, the *de novo* synthesis of larger parts or the whole genome becomes more economic [71]. The synthesis of whole genomes comes with a higher workload and economic cost, but its yields may outcompete the genome capture and large-scale genome engineering processes [71]. *De novo* genome synthesis allows for a much higher degree of freedom in terms of the design of the actual chromosome, such as the recoding of multiple codons at once, and yeast has been proven to be the best chassis to date. Additional recent research has shown that even a functional RNA genome can be generated by first assembling a DNA genome *in yeasto* and subsequent in vitro RNA production to generate a naked RNA genome which can be used for the transfection and production of functional viral particles [67]. Based on their rather small genome sizes, viral genomes can be built rapidly and many viruses have already been built [27]. The ability to quickly build infectious viruses has many advantages for humanity, such as functional characterisation of unknown virus variants even before they appear in nature. This has a huge advantage over the functional characterisation of individual protein libraries because the virulence and pathogenicity of the synthetic viruses can be tested in cell culture, allowing for the generation of in vivo data. Subsequent characterisation of virus variants would allow researchers to quickly identify highly relevant genome regions for virulence and pathogenicity, resulting in efficient targeting of those regions for drug and vaccine development. Such an approach may allow for the generation and testing of drugs and vaccines before an outbreak happens, which would allow for a fast response. Preempting outbreaks thus might prevent a pandemic situation, such as that caused by SARS-CoV-2 which, while ongoing, has already caused significant economic damage in all areas of life [72]. However, these technical developments need to be carefully monitored by competent authorities and must be conducted in a transparent way to prevent misconduct. The dual-use character of this technology is rather extreme: on the one hand, it bears immense knowledge gain, but on the other, it has risks which are constantly discussed in great detail, such as in the context of the resurrection of the extinct horsepox virus [73,74,75,76,77]. 

Besides all the advances and the biosafety aspects, both strategies, heterologous genome capturing and *de novo* synthesis, share a major bottleneck, which is the extraction and transfer of large DNA constructs from yeast. This process is called genome transplantation and has to date only been successful in a few cases [64,78]. One should always keep in mind that a heterologously constructed synthetic chromosome is not necessarily functional, and the lack of candidates with transplanted genomes does not give a clear indication of whether the chromosome is unable to support life or if the transplantation itself is the problem [79]. So far heterologous genome transplantation has only shown limited success and seems to be highly dependent on the recipient cell abilities. *Mycoplasma*, which was the first organism ever reported to have a genome transplanted, might be an ideal host because of the lack of a cell wall. This indicates the need for alternative chassis, ideally with already established tools which could, for example, allow for the transfer of engineered or synthetic chromosomes via interspecies and interkingdom conjugation such as *Sinorhizobium meliloti* [80,81]. Currently, a chassis which has all the benefits of the *S. cerevisiae* genome engineering and the synthetic genomics toolbox plus the ability to perform interspecies and interkingdom conjugation is still lacking. Alternatively to whole chromosome assembly and synthesis, the development of larger fragments in vitro or *in yeasto* has advantages. The partial replacement of a given genome with larger synthetically generated sequences gives direct fitness readouts, which allow for the simple and fast pinpointing of problematic sequences and circumvents the bottleneck of the genome transplantation procedure [22,26].

In addition to using *S. cerevisiae* as a chassis for the construction and engineering of heterologous chromosomes, yeast is on the path to becoming the first synthetic designer eukaryote, as an international consortium is aiming to build synthetic yeast 2.0 [21,82].

## 5. Yeast 2.0: Building the First Synthetic Designer Eukaryote 

**Figure 2:**
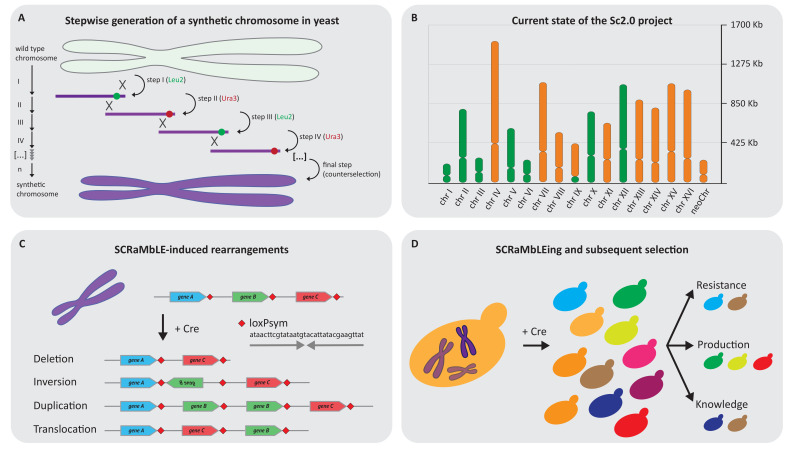
The Sc2.0 project and its applications. (**A**) Step by step replacement of Sc1.0 DNA by chemically synthesised DNA to generate Sc2.0. Marker swapping allows for an efficient replacement of Sc1.0 DNA and gives a direct readout if Sc2.0 DNA is causing a phenotype. It is critical that the penultimate fragment contains the Ura3 marker to be able to perform counterselection with 5-fluoroorotic acid (5-FOA) to ultimately generate a marker-free strain. (**B**) The current state of the Sc2.0 project. Green indicates the finished and published chromosomes, whereas the chromosomes which are being finalised are orange. The graph also indicates the 17th nuclear chromosome which is the tRNA neochromosome containing all 275 nuclear tRNA genes. Note: for chromosome IX, only the right arm is synthesised and chromosome I is reported on the bioRxiv preprint server. (**C**) The possible outcomes of Cre recombinase on two symmetrical loxP (loxPsym) sites (deletion–inversion–duplication–translocation) indicated via gene B (green). Translocation can occur within one or between multiple synthetic chromosomes, with only the intrachromosomal event depicted here. (**D**) SCRaMbLE is an accelerated evolution tool for applied and basic sciences. Induction of Cre in synthetic yeast strains generates a highly complex population which can subsequently be screened for the desired phenotype, which can be used to answer basic questions or boost biotechnology applications.

Synthesising a 12 Mb genome is an extraordinary scientific endeavour and might be one of the biggest efforts in science since the sequencing of the human genome [82] (Figure 2A,B). The first partially synthetic yeast chromosome was published in 2011, presenting the synthetic right arm of chromosome IX, synIXR [83]. However, the idea was born earlier and intensive planning was performed before the physical start of the Sc2.0 project. The Sc2.0 project aimed to not only be a redesign of the natural yeast genome; multiple design rules were implemented and applied to the whole yeast genome [21]. Perhaps the most significant of these, at least from the applied perspective, could be the integration of symmetrical loxP sites (loxPsym) 3 bp downstream of every non-essential gene [83]. Upon the expression of Cre recombinase, the synthetic chromosome(s) are like a deck of cards and extensive genome alterations are possible: the cards can be shuffled, turned around and added to or removed from the deck (Figure 2C). This system is termed *Synthetic Chromosome Rearrangement and Modification by LoxPsym-mediated Evolution* or, in short, SCRaMbLE [83,84]. Another important alteration is the removal of all 275 nuclear tRNA genes from their native loci, followed by their relocation on a neochromosome, the 17th nuclear chromosome in the Sc2.0 strain [21,85]. One of the main reasons for this is to create a more stable yeast genome, as tRNA genes are known hotspots for genomic instability, e.g., by head-on collision of DNA replication and transcription machinery or via the preferred transposon integration upstream of tRNA genes in yeast [86,87,88,89,90]. Stably integrating additional chromosomes into the nuclear genome of yeast has a huge potential and the Sc2.0 tRNA neochromosome may pave the way to deeper understanding of how extraordinarily complex constructs can be built and maintained as additional chromosomes.

The physical start of the Sc2.0 project may have been the launch of the “Build-a-Genome Course” at Johns Hopkins University in 2007, where undergraduate students learned to assemble oligonucleotides within the Sc2.0 project into larger fragments [91]. Obviously, the whole yeast genome project could not be solely conducted by a single, student-driven effort, therefore an international consortium was formed and the synthesis of the individual chromosomes was distributed to individual research groups working to the same design rules [21]. The synthesis effort includes research institutions from five countries across four continents, with numerous other countries involved in collaborative efforts to characterise the synthetic chromosomes [82]. It is impressive that it will presumably take only around 10 years for the synthesis and characterisation of all 16 chromosomes plus the additional tRNA neochromosome, which is equivalent to building roughly one 1 Mb *Mycoplasma* genome per year! The progress, starting from the right arm of one chromosome (synIXR) in 2011 [83] to the first fully synthetic yeast chromosome (synIII) in 2014 [92], followed by the synthetic chromosomes synII, synV, synVI, synX and synXII in 2017 [93,94,95,96,97], leads to the strong impression that the remaining 9.5 synthetic chromosomes, as well as the additional tRNA neochromosome, will be finalised in the near future [82,85] (Figure 2B). To efficiently build the entire synthetic 12 Mb genome of *S. cerevisiae*, each of the chromosomes is constructed in a separate haploid strain, allowing for in parallel construction of the individual chromosomes, followed by consolidation of all synthetic chromosomes within a single cell via subsequent rounds of mating and sporulation [21]. To maximise the efficiency of chromosome consolidation, the wild type counterparts of the synthetic chromosomes are destabilised in the respective diploid semi-synthetic strain. Destabilisation leads to the loss of the targeted wild type chromosomes, omitting cross-over effects of synthetic and wild type chromosomes during meiosis. The destabilisation is achieved by either integrating a strong inducible promoter upstream of the centromere or by targeting the centromere with CRISPR/Cas9, which ultimately leads to the loss of the targeted wild type chromosome(s) by the generation of an n-1 aneuploidy [94,98]. Subsequently, sporulation results in haploid semi-synthetic strains of both mating types with multiple synthetic chromosomes, which are the starting point for further rounds of synthetic chromosome consolidations until the fully synthetic strain is obtained.

The whole community is anticipating the final Sc2.0 strain with all synthetic chromosomes consolidated in a single cell. The Sc2.0 strain will indeed be an outstanding new synthetic biology tool, the significance of which will be unravelled in the future. However, initial pilot studies with semi-synthetic yeast strains have already shed light on the potential of the final Sc2.0 strain.

## 6. Yeast 2.0 Lessons and Application for the Future

The Sc2.0 project shows the plasticity of the yeast genome. The genome can be altered to a high degree without showing a drastic change in the phenotype under various conditions. However, whether the final Sc2.0 strain with all synthetic chromosomes in a single cell will have a wild type-like phenotype remains to be seen. Semi-synthetic strains with multiple synthetic chromosomes have been generated and shown to have a wild type-like phenotype, which is a promising sign for the final Sc2.0 strain. One interesting finding in the synthetic chromosome 12 (synXII) strain is that the rDNA repeat can be removed from its natural location and the rDNA can be supplied solely from a multicopy plasmid [93,99]. The nucleolus structure in this strain is disrupted but has no impact on the growth of the respective strain. Strikingly, relocating the rDNA locus to a single locus on different chromosomes restores the natural nucleolus structure with only minor effects on fitness. This result indicates that the organised nucleolus structure in yeast relies simply on the tandem structure of the rDNA repeat and not necessarily on its chromosomal location. However, if multiple locations for the rDNA are present, such as in the case of the multicopy plasmid, the structure of the nucleolus might be altered.

Potentially, one of the most intriguing features of the Sc2.0 genome is the implementation of SCRaMbLE [21,83,100] (Figure 2C). In a recent series of articles, the power of this system was demonstrated for strain optimisation and to boost the production of compounds such as carotenoids, penicillin and violacein [101,102,103] (Figure 2D). On one hand, this can lead to new discoveries to optimise fluxes within cells to be more efficient producers or it can alter the genotype in such a way that a strain has an increased fitness or stress tolerance [104]. However, one should always keep in mind that there are limitations—even with SCRaMbLE—because loxPsym sites are only 3 bp downstream of every non-essential gene. In the case of overlapping genes or essential genes, there is no loxPsym site, which results, in certain cases, in the connection of multiple genes which can only be SCRaMbLEd in tandem, reducing the degree of freedom and resulting in instances where non-essential genes are coupled with essential genes. This may result in the possibility that the optimal genotype in a certain SCRaMbLE experiment cannot be obtained. However, there is a new approach to circumvent this limitation by concatenating the essential genes of the synthetic chromosome on a “non-SCRaMbLEable” centromeric plasmid [105]. This additional copy of essential genes now allows for the loss of the essential rafts of the synthetic chromosomes and increases the number of combinatorial possibilities which can be obtained by SCRaMbLE. SCRaMbLE itself is the driving force to develop new techniques, such as a tightly light-regulated Cre recombinase or reporter systems to increase the fraction of cells where Cre recombinase is active [106,107]. The reporter system is especially interesting in cases where no simple readout, such as higher tolerance or a more or less intensive colour, can be obtained. Interestingly, SCRaMbLE was used to improve the production of betulinic acid, but betulinic acid is a compound which has no simple readout. To circumvent this challenge, the Ellis lab developed an elegant semi-automated sample preparation pipeline coupled to a rapid LC-MS screening for betulinic acid [108]. This workflow was able to identify strains with up to seven-fold increased production after screening 1000 candidates. 

With the Sc2.0 project nearing its completion, a new powerful yeast chassis will be available to the research community and broad applications are guaranteed [85]. The Sc2.0 project might be cited as the project which has been a main driving force for advances in DNA synthesis. The DNA synthesis field is evolving and alternatives for the chemical DNA synthesis by enzymatic DNA synthesis may open new horizons—one can envisage benchtop synthesisers in many laboratories [20,28,85]. These prospects open completely new scales for genetic engineering approaches and one can ask: what have we learned from Sc1.0 and Sc2.0 and can this be implemented inSc3.0?

## 7. Yeast 3.0: What Could Be the Future Design Approach?

As with every piece of software, there may be a couple of smaller changes to Sc2.0 before there is a complete upgrade to the next version, Sc3.0. Intermediate versions will remove initial “bugs” in the software and come at a generally lower cost before the whole software gets a remake. Intermediate versions, where the synthetic genome is reduced, altered or extended according to the Sc2.0 rules or with new, additional rules, have great potential for future application. How Sc3.0 would look remains unclear, however, recently, a perspective article by Junbiao Dai and others [105] pointed out a minimised version of yeast with major changes in the genetic code might be the Sc3.0 project. One should keep in mind the intermediate versions from Sc2.0 to Sc3.0 have the potential to answer questions in basic and applied research. The design of future versions is only limited by the creativity of the designer and the basic principles of life. The beauty of synthetic genomics is that it is turning into a field of “creative writing”, as synthesis costs are dropping and recent developments allow for the construction of whole chromosomes. In this section, recent research is discussed which might have an influence onSc2.X to Sc3.0 and beyond.

### 7.1. Varying the Number of Chromosomes and Their Confirmation

**Figure 3:**
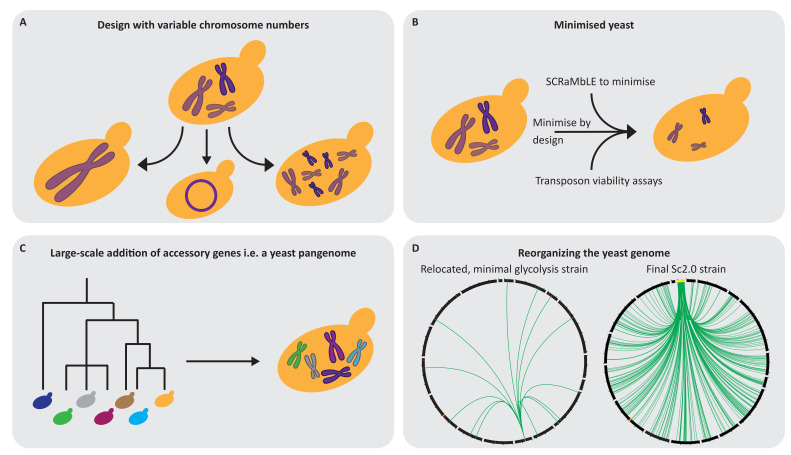
Potential design principles for the Sc3.0 genome. (**A**) Recent data suggest that Sc3.0 can be completely free in its design in terms of the number of chromosomes and may be only a single circular chromosome. (**B**) Sc2.0 has the potential to be the foundation to achieve a minimal genome. The interplay of a minimised Sc2.0 design, subsequent SCRaMbLE and functional dissection (i.e., characterisation via transposition) could result in a truly minimised Sc3.0 genome after multiple rounds. (**C**) The Sc2.0 genome is based on the *S. cerevisiae* S288c strain, however, the genome content between different *S. cerevisiae* isolates shows large variations which have potential for new properties if transferred into the SCRaMbLEable format of Sc2.0. The potential Sc3.0 project should consider building a larger genome which could consist of an *S. cerevisiae* pangenome or a highly diverse “yeast chimera”. (**D**) Recent studies realised or proposed the functional clustering of genes in yeast for subsequent biological studies. The circles represent the whole genome of two studies: depicted on the left is the relocation of a minimal glycolysis pathway in Sc1.0 [116] and, on the right, the tRNA relocation within Sc2.0 [21]. The plots start with chromosome I at 12 o’clock, with clockwise representation of the remaining chromosomes, including the mitochondrial chromosome. Individual chromosomes are indicated by black lines, except for the tRNA neochromosome, which is yellow. Green lines indicate a relocation of either a glycolysis gene (left circle) or tRNA gene (right circle). Red marks in the left circle indicate deletions in the genome. The orange bar in both circles indicates the repetitive rDNA location, which is the only exception to the scale. The indicated scale bars are 250 kb steps. Plots in D were generated with circos [117].

*S. cerevisiae* has 16 linear, nuclear chromosomes, while other species, such as *Schizosaccharmoyces pombe,* for example, have only four nuclear chromosomes. These chromosomes also differ in their type of centromeres: *S. pombe* chromosomes have the common type of regional centromeres, while *S. cerevisiae* and related budding yeast species have a rather unique type of short point centromeres [109,110]. Yeast species with point centromeres usually have a greater number of chromosomes compared to yeasts with regional centromeres. This could be interpreted as an implication that larger regional centromeres are required for efficient segregation of large chromosomes. Interestingly, this hypothesis was proven to be wrong. Strikingly, the alteration of chromosome numbers to a single chromosome and even its subsequent circularisation can be achieved in *S. cerevisiae* [111,112,113]. This indicates that a potential Sc3.0 project would not need to stick to the general karyotype of *S. cerevisiae* and can be completely free in its design (Figure 3A). On the other hand, it seems that the yeast genome is capable of containing many more chromosomes, which indicates that yeasts with much more than 16 chromosomes can be generated [114,115]. These additional chromosomes are currently termed neochromosomes and the tRNA neochromosome of the Sc2.0 project is one of the examples which shows the creativity which one can implement in synthetic genome designs [21,85].

### 7.2. Creating a Simplified Yeast Genome

It seems that genome duplication in *S. cerevisiae* is a common event which allows for quick stress adaptation [118,119,120,121]. It is accepted that an ancestor of *S. cerevisiae* underwent a whole genome duplication and subsequent diversification into many different species [122,123]. Many genes have redundant counterparts in the *S. cerevisiae* genome, which would potentially allow for a strong “simplification” of the genome (Figure 3B). Generating streamlined yeast strains would potentially result in a simpler metabolic model and better prediction of fluxes. It would allow for a more controlled design to optimise production of valuable compounds or implement pathways to understand the fundamentals of life and would make computational modelling and prediction feasible. Ultimately, this leads to the question of which the core genes are that are necessary to produce viable cells. Recent work has shown that a bacterial genome of 531 kb can be built, containing 473 genes sufficient to allow the cells to grow, which represents a reduction of the ancestral genome by ~50% [65]. Interestingly, a bottom-up approach by the same group based on a *de novo* designed minimal genome was not successful. Research in *S. cerevisiae* is still far from generating a minimal or essential yeast genome. Many functional studies have harnessed the power of the single and double deletion collections and generated a massive amount of data on different interaction networks [124,125,126,127,128]. Functional profiling of the *S. cerevisiae* genome indicated that around 20% of the yeast genes are essential under rich media conditions, although the deletion of two genes classified as non-essential in a single cell can have a lethal outcome [126,128]. All obtained knowledge and data would still not be sufficient to generate an essential yeast genome in a bottom-up procedure. Notably, the combinatorial effect of deletions might also restrict the generation of a minimal yeast genome in a top-down approach. However, the Sc2.0 project and its implemented SCRaMbLE system potentially allows for some progress towards a minimal yeast genome. Based on the stochastic effect of SCRaMbLE, there will not be a single minimal genome but many different versions. Large-scale analysis of yeast genome variations, in combination with quantitative trait loci (QTL) analysis, may allow researchers to gain more understanding about the core genome of *S. cerevisiae*. The aforementioned limitation of non-essential genes coupled to essential genes could be bypassed by an essential chromosome. This approach may allow for the construction of an Sc3.0 minimal genome in the future, as proposed by Junbiao Dai et al., but it remains unclear how many different “minimal” variants would be the result of such an approach.

### 7.3. Implementing New Functions for Subsequent Adaptation

Clearly, a minimal genome is highly interesting. However, on the other hand, diversity afforded by non-essential genes is highly valuable. There are many different *S. cerevisiae* isolates and the genome content differs drastically, it would make sense to implement this in a new genome design [129,130] (Figure 3C). There are wild yeast strains which have certain benefits or strains which have been adapted over many generations to a certain process, such as specific strains for beer and wine fermentation. In particular, traditional Norwegian farmhouse brewing strains seem to have some interesting capabilities, such as high temperature tolerance and fast fermentation capabilities [131]. Inclusion of all additional known yeast genes not present in the Sc2.0 genome in a SCRaMbLEable pangenome neochromosome would potentially allow for higher flexibility of SCRaMbLEing yeast towards biotechnological applications. Lessons learned in this strain background have the potential to be transferred to industrial strains to boost biotechnological applications. From the pangenome, one could argue for generating species chimeras on a larger scale and not being limited to an *S. cerevisiae* pangenome. Currently, attempts of the introduction of heterologous genes into a host organism are in the lower numbers, however, technologically it is now possible to construct DNA on a megabase scale, and hundreds, if not thousands, of genes can be additionally incorporated into the yeast core genome. Initial work in yeast shows that even a humanisation of yeast is feasible. An interesting work points out that the histones of *S. cerevisiae* can be swapped for human histones, and yeast cells are viable after a prolonged adaptive process [132].

### 7.4. Changing the Genomic Landscape

Research has made enormous progress in reading DNA: whole-genome sequencing became a standard technology and is accessible for every molecular biology lab. However, the structure and the higher order of genomes is still not fully understood. Technologies such as Hi-C, a chromosome conformation capture variant, allow for the 3D and even 4D visualisation of chromosomes and gain insights into chromosomal biology. However, there is still a lack of knowledge as to how gene order influences the genome structure. One question which arises is: why is the yeast genome less ordered compared to a bacterial chromosome? The operon structure in bacteria often clusters functional genes together, while this is usually not the case in eukaryotes [133]. Would it be feasible to reorder the yeast genome to allow systematic characterisation of pathways or genome structure-influencing elements? In an initial study showing that it is feasible to do so, a group of researchers successfully generated a strain with a minimal carbon metabolism and ordered these genes on one distinct locus in the yeast genome [116] (Figure 3D). This study presents the largest relocation of the yeast genome to date and opens new opportunities of pathway swapping, which will be beneficial to further optimise yeast as a production chassis. The Sc2.0 tRNA neochromosome is a similar but much bigger effort, with the relocation of 275 highly expressed tRNA genes with complex sequence homologies. The progress in these projects will confer new insights into cell physiology and will give new lessons for the creative writing of genomes.

The number of genome designs and their respective variation are almost unlimited: their only limitation is that they must be able to support life. Interestingly, in recent years, research towards the incorporation of non-natural amino acids and non-natural base pairs has improved drastically [134,135,136,137,138]. The latter has to date not been transferred to yeast and initial work has been done in *E. coli*, but it is impressive that researchers are able to perform such drastic changes in the genome. However, the incorporation of non-natural amino acids is becoming an interesting tool and may benefit from synthetic genomes which have reduced numbers of codons. Xenobiology will open a completely new toolbox of applications and concepts for synthetic genomics, and it will be possible to implement another layer of security in genetically engineered and synthetic organisms, which minimises the risk of environmental contamination, such as has happened with genetically engineered mosquitoes [139].

## 8. From Yeast 1.0 to Yeast 3.0: Summary and Future Perspectives

Yeast has been an important model organism for more than 40 years and “an experimental organism for 21st century biology” [34] and will continue to be critical beyond this. Without the fundamental work on Sc1.0 and its application in biotechnology, humankind would not have reached the level it has. The Sc2.0 project is nearing its completion and has already generated a massive gain in knowledge, new techniques and proposes new concepts for the field of synthetic genomics. What is the potential for the next generation of yeasts? “*Evolution does not produce novelties from scratch. It works with what already exists*” is a fitting quote from François Jacob in 1977. Human curiosity and technological advances now allow for the large-scale engineering of microbes and implementation of complex heterologous functions to enhance evolution. Genetic engineering is the basis to achieve this and its application has the opportunity to tackle many challenges of humankind and increase living standards. Modern synthetic biology and synthetic genomics approaches open new horizons in research, breaking the limits of science down to the creativity of the scientist. There are many visionary researchers doing outstanding research to improve our everyday lives, and today we need to consider sustainability as one of the most important factors. It would be amazing if in the future, biotechnology could be done with a photosynthetic yeast, or at least a yeast which is able to utilise CO2 or formate rather than glucose as a carbon source. The first steps towards such a yeast are being investigated [140,141,142]. Genetic engineering would allow researchers to equip yeast with new sets of enzymes or even organelles and give evolution the power to work with novelties, which may make those visions reality.
